# A Case of Angina Pectoris

**Published:** 1884-03

**Authors:** P. W. Van Peyma


### A Case of Angina Pectoris.

Reported by P. W. Van Peyma, M. D.

On the night of December 3, 1883, I was called by a midwife to attend Mrs. M., aged 27 years, in her first confinement. The position of the child was occiput posterior, with the head well engaged. The delivery required the use of forceps, and was accomplished with considerable difficulty. The child was dead. After delivery a tumor, the size and consistency of an ordinary orange, was observed to the left of the fundus uteri. The connection of this tumor with the uterus was not established. The midwife informed me that she had remarked its existence two or three months previously. Subsequent to the parturition, during a period of about ten days, the patient suffered from suppression of lochia, general abdominal tenderness, high temperature, etc. At the end of two weeks, however, she was restored to ordinary health, the tumor having also disappeared. During an interval of three weeks she continued in good health, attending to household duties and making occasional visits to her friends. Early in the morning of February 5th, the lady's husband called at my office, saying that his wife had been ill since the previous evening and was suffering severely from pain in the region of the stomach. I prescribed opium and belladonna in moderate doses and promised to call later in the day. My visit was made about 11 A. M. The patient, I was informed, had taken two or three doses of the medicine prescribed, but had, so far from being relieved, become much worse. On reaching the bedside all present, including myself, were astonished at her appearance, and I believed that she was dying. The lips were pale. The pulse and heart-beat could not be determined. The respiration was limited to an occasional slight gasp, any one of which I thought might be the last. The eyes were glassy and insensible to the touch of the finger. The extremities were cold. After having remained at the bedside about ten minutes, I noticed that the respiration had become a little more frequent and regular, and some effort was made towards reviving the patient. After another interval of ten minutes the cardiac sounds were faintly heard, (the action of the heart being regular and unaccelerated,) and later these were determined to be perfectly normal. At this time commenced recurring spasmodic expiratory cries. The pulse now became perceptible and the general condition improved so rapidly that upon leaving, an hour later, I was confident of speedy recovery. I may here say that my diagnosis at this time was hysteria, though I was obliged to admit that I had never seen a case so severe.

My next visit was made about 1 P. M. The patient was so far restored that she conversed freely and rationally with me regarding the symptoms experienced since the previous evening. In her own words : "I was preparing supper, feeling quite well, when suddenly it seemed as if something flew right to my breast, and immediately I felt a severe pain in the stomach and breast. This lasted all night with hardly any interval of comparative ease." I was informed by her, as well as by her acquaintances, that she had never experienced any similar ailments, having, however, been frequently sick with various diseases, such as variola, typhoid fever, etc. At this visit nausea was the only remaining indication of ill-health, and I considered the patient as entirely out of danger and practically recovered. The husband reporting later in the afternoon that he had been unable to obtain the urine, which had been requested for the purpose of analysis, I promised to call about 5 P. M. Upon reaching the house, shortly before six, I learned that the patient had had a recurrence of the symptoms of the morning, and with fatal results, she having died only a few minutes before my arrival. An autopsy was refused.

In the light of all the events I have no hesitation in considering the case as one of angina pectoris. The paroxysmal nature; the character and situation of the pain; the spasmodic, or perhaps forced, character of the respiration as consciousness returned, expressing a sense of suffocation; the pinched and anxious features ; the pallor of countenance; the coldness of extremities ; and the condition of the heart, all point towards that obscure disease, or group of diseases, known as angina pectoris.

And yet, as has been remarked, the case was, at one of its stages, mistaken for one of hysteria. In numerous particulars, too, the case presents exceptions to the rule. According to the statistics of Sir John Forbes, reported in "Reynolds' System of Medicine," of eighty-four cases, only eight were in women. Also is the case an exception to the rule laid down by the same authority in that the majority of the cases occur in connection with the gouty diathesis, and in that "angina is an attendant rather of ease and luxury than of temperance," "on which account it is more frequently met with among the rich, or in persons of easy circumstances."

In this connection we wish also to call attention to the leading article of this number of the JOURNAL on glonoin, or nitroglycerine, and to a paper published in this JOURNAL in 1881, written by Dr. H. R. Hopkins, on the subject of angina pectoris in its relation to arterial tension, as studied by the sphygmograph.

